# What’s to Eat and Drink on Campus? Public and Planetary Health, Public Higher Education, and the Public Good

**DOI:** 10.3390/nu15010196

**Published:** 2022-12-30

**Authors:** David Arthur Cleveland

**Affiliations:** Environmental Studies Program, Department of Geography, University of California, Santa Barbara, CA 93110-4160, USA; cleveland@ucsb.edu

**Keywords:** diet and health, food environments, food justice, food security, neoliberalism, planetary health, public health, public higher education, sufficient consumption, sustainable consumption

## Abstract

Public higher education institutions (PHEIs) have a unique and important role in responding to the public and planetary health crisis—they are centers of research on public and planetary health and of learning for young people, and have a public good mission. Yet, PHEI campus food environments are predominantly unhealthy and environmentally unsustainable, and associated with unhealthy food choices and unhealthy students. PHEIs are addressing high levels of student food insecurity (FI) that disproportionately affect the most vulnerable groups. Yet, because student FI is measured as individual access to adequate quantities of food, campus responses to FI often overlook unhealthy food environments. These environments result from neoliberal PHEI business policies that prioritize short-term revenue and encourage superfluous consumption, and unhealthy, environmentally harmful diets. PHEIs need to move beyond neoliberalism to honor their public good mission, including prioritizing health, the environment, and equity, in decisions about food on campus. My goal in this perspective is to encourage inclusive campus discussion about why this change is required to adequately respond to the crisis of student, public, and planetary health, and about how to begin.

## 1. Introduction

**Figure 1:**
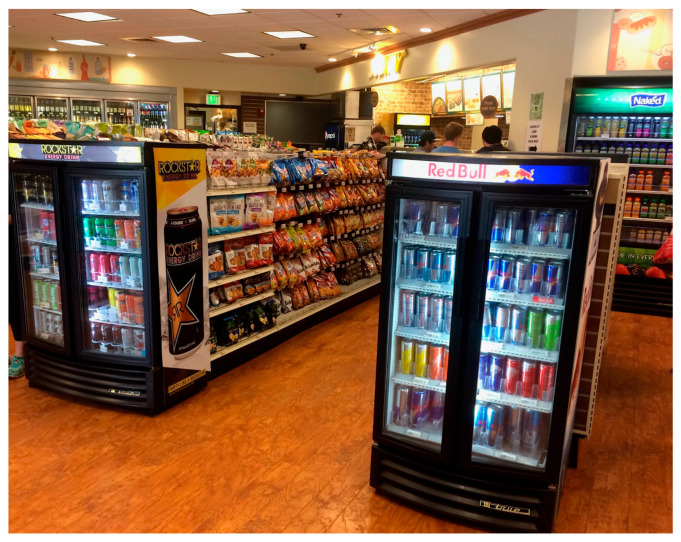
Convenience store on a PHEI campus. Credit: © D.A. Cleveland, used with permission.

When I walk across my public university campus I see a food environment dominated by national fast food franchises, and vending machines and convenience stores stocked mostly with sugary beverages and unhealthy foods. The diets this kind of food environment encourages are fueling the high and rising prevalence of obesity, and noncommunicable diseases (NCDs), such as diabetes and heart, liver and dental disease. Many of these foods, especially meat and dairy, are also a major cause of environmental destruction, climate change, biodiversity loss, and economic inequity [1]. While these foods and beverages are prominently displayed, they are not accompanied by information to help students make food choices based on environmental or social impacts. Yet, the campus has food programs that support health and the environment, although not visible in my walk, including purchasing fresh local produce for residential dining halls, a campus food garden, a food pantry, and classes on purchasing and preparing nutritious food. The campus also has policy statements of its public-good mission and its commitment to public and planetary health. My campus food environment and programs are similar to those of public higher education institutions (PHEIs) in the US, and increasingly in the world (Figure 1).

These environments result from neoliberal PHEI business policies that prioritize short-term revenue, and encourage superfluous consumption and unhealthy, environmentally harmful diets that negatively affect students’ health, food security and academic success, and impact marginalized groups disproportionately [2,3,4]. The conflict of these policies with PHEIs’ public-good mission of promoting health, food security, equity, and academic success compromises PHEI integrity. It also means that campus staff and students are constrained in their efforts to create more healthy, environmentally sustainable, and equitable campus food environments.

Understanding this conflict has become more important for me with my involvement in some of the University of California’s system-wide programs—the Healthy Campus Network, the Research Consortium on Beverages and Health, and the Healthy Beverage Initiative (HBI). For example, the HBI has goals of decreasing sugar-sweetened beverage (SSB) consumption and increasing tap water consumption on the UC’s 10 campuses to improve health and decrease negative environmental impacts [5]. Yet, these goals conflict with the pouring rights contracts with either PepsiCo or Coca-Cola that nine of the campuses have, which are supported by campus business policies in order to increase revenue. These contracts obligate campuses to partner with these corporations in promoting consumption of SSBs and other commercial beverages, whose negative effects on health and the environment have been well documented [6].

PHEI campus food environments are part of the neoliberal global food system that is dominated by industrial agriculture and multinational food corporations—a system that promotes increasing production and consumption of environmentally destructive unhealthy food and beverages [7,8,9]. This system is a key driver of the unprecedented public and planetary health crisis—the pandemic of obesity and diet-related NCDs, and the negative impact on the environment, biodiversity, climate, and economic equity, that threatens health and the supply and accessibility of food itself [10,11].

To adequately respond to this planetary and public health crisis and avoid catastrophe, scientific research increasingly supports the need for radical transformation of our relationship with the Earth and with each other. This includes demand-side solutions to reduce net human impacts by moving away from economic growth, and by reducing superfluous consumption in wealthier populations, such as those in the US, so that resources can become available to under-consuming populations [12,13,14,15], which can also increase well-being in over-consuming populations [16]. These transformations are just beginning to be advocated, for example, by the IPCC (The Intergovernmental Panel on Climate Change) [17] and the European Union [18], although still far from having a major policy impact.

Reducing consumption of unhealthy environmentally destructive food, especially ultra-processed food and meat and other animal source foods that have relatively large negative environmental and/or health impacts, is a key component of successfully responding to the crisis [1,8,19,20]. When health and environmental impacts are included, these changes can also reduce costs. For example, a modeling study found that healthier, more climate friendly, plant-based diets, along with associated reductions in the costs of healthcare and climate change, can reduce the cost of food over time, not only in high-income countries, but in low-income countries as well [21].

As institutions with a public-good mission [22,23] and centers for research in health, environment, food, and agriculture, PHEIs globally have a unique and important role to play in these needed changes. They also have a direct influence on the majority of young people in wealthier, over-consuming populations such as those in the US—in 2020 almost 20 million graduate and undergraduate students attended higher education institutions (HEIs), comprising 74.5% of 18–19-year-olds and 40.6% of 20–24-year-olds in the population, with 73.7% of all students at PHEIs [24].

In this perspective I ask “How can we understand and resolve the contradiction between PHEIs’ public good mission and their unhealthy, environmentally unsustainable campus food environments in the context of the public and planetary health crisis?” To answer this question, I address more specific questions, focusing on the example of the US—“How do campus food environments affect student food choice, food security, and health?” “Why are campus food environments unhealthy and unsustainable?”, and “How can we transform campus food environments commensurate with the unprecedented challenges of the public and planetary health crisis?”.

## 2. How Do Campus Food Environments Affect Student Food Choice, Food Security, and Health?

The food environment, including physical, economic, informational, and sociocultural components, is one of the key variables influencing individual food choices [25,26], and therefore, diets’ health and environmental impacts. This is especially important for college students because most are at a critical age for the development of dietary knowledge, attitudes, and habits that can persist long after graduation [27], and affect health and the environment in later years [28,29,30].

### 2.1. Campus Food Environments and Student Food Choice

**Figure 2:**
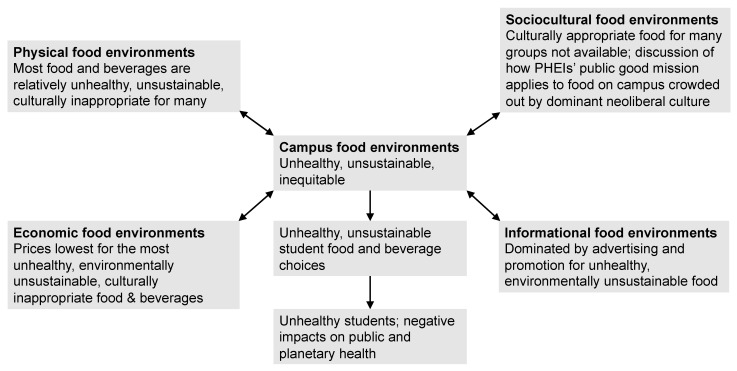
PHEI campus food environments, student food choice, and health. © D.A. Cleveland, used with permission.

The food environment is the main variable affecting food choice that PHEIs have direct control over, and food environments on and near PHEI campuses are generally unhealthy in the US [31,32,33] and in many other countries [34], e.g., Australia [35] and Brazil [36] (Figure 2). Focus groups at US PHEIs have found that students themselves believe that campus food environments are culturally inappropriate and that physical and economic food environments encourage choosing unhealthy foods [37,38,39]. A survey of 1149 first-year students at 8 HEIs (including 7 PHEIs) found that as the importance of price (economic environment) increased in student food choices, consumption of fruits and vegetables decreased and consumption of sugar-sweetened beverages (SSBs) and added sugar increased, and as the importance of advertising (informational environment) increased, consumption of SSBs and added sugar increased [40].

It is, therefore, not surprising that students’ food choices both on and near campus are also generally unhealthy. Most surveys have found that student purchases of food on campus (excluding dining halls) and near campus were associated with unhealthy foods, such as SSBs and fast foods high in fat and added sugars [41,42,43]. A survey of 209 first-year PHEI students found that 64% considered their eating habits off campus healthy, compared with only 56% on campus [44].

### 2.2. Student Food Security, Food Choice, and Health

**Figure 3:**
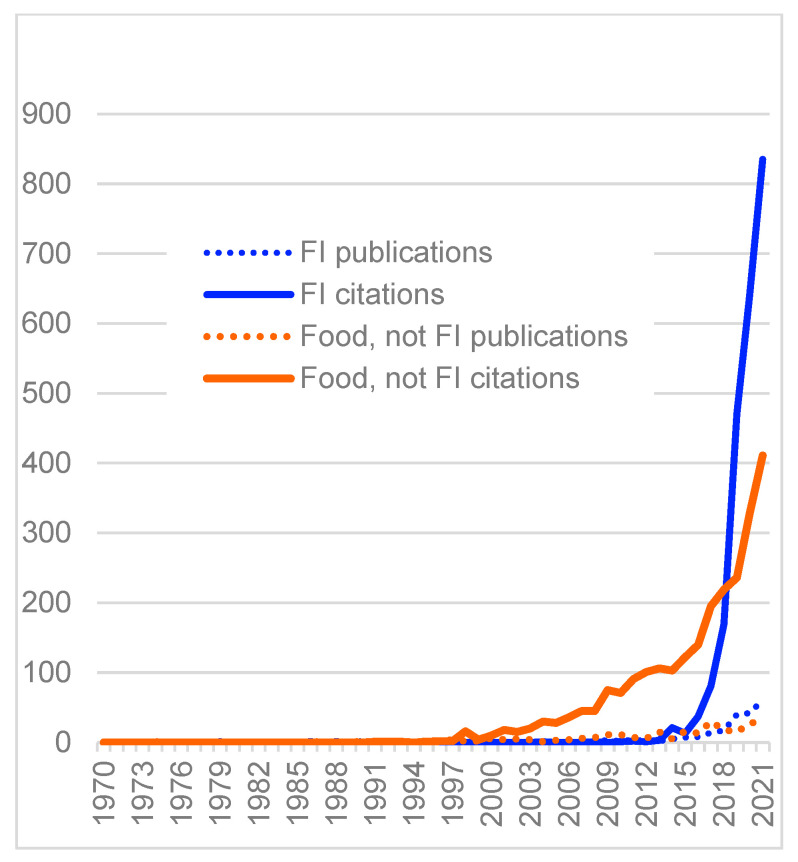
Trends in publications and citations for food insecurity (FI), and for food environment, food choice, diet, and health, on college and university campuses. © D.A. Cleveland, used with permission. Data from Google Scholar search of title terms, 9 April 2022: **FI**: ((food security) or (food insecurity) or hunger) and ((higher education*) or universit* or colleg* or campus*); **food, not FI**: (food* and (environment* or choice* or diet* or health*)) and ((higher education*) or universit* or colleg* or campus*) not ((food security) or (food insecurity) or hunger).

While there are other indicators of diet-related health, both more direct (e.g., overweight, obesity, and NCDs), and indirect (e.g., type of foods eaten and the food environment), food security is the indicator that has increasingly dominated research, discussion, and action about diet-related student health at HEIs, for example, as shown by published articles and citations (Figure 3).

The standard instrument for defining and measuring food security in the US is different versions of the USDA’s U.S. Household Food Security Survey Module, focused on the adequacy of the quantity of food accessible as reported by respondents [45], and has been a basis of a popular global survey instrument [46]. Many surveys in the US have found student food insecurity (FI) prevalence of up to 40% or more [47,48,49], much higher than in the general population. In response, many policies and programs have been created to reduce FI on campus [50,51] and, given the acute nature of FI, their focus is understandably on mitigating its short-term, proximal causes by increasing the quantity of food available to FI students.

However, food security is not a good indicator of diet-related student health because it does not include diet quality, so that being food secure does not equate with adequate nutritional intake. Although the USDA food security definition includes “ready availability of nutritionally adequate” food, the only reference to this in the full 18-item Survey Module is two undefined mentions of “balanced meal” [45], and campus FI assessments often used abbreviated versions of the USDA survey, which contain only one or no mention of “balanced meal”. As a result, nutrition is often “overlooked or disregarded” in food security assessment [52], and nutrition security has been proposed to replace food security in order to emphasize food quality over quantity, but that also does not include food environments [52,53].

**Figure 4:**
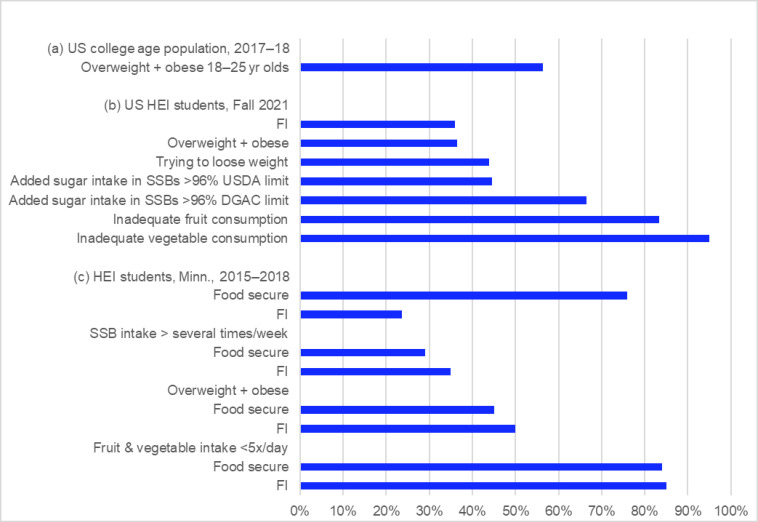
Health of US higher education students. © D.A. Cleveland, used with permission. (**a**) Data from [54] for NHANES sample, *n* = 383. (**b**) Based on data from the National College Health Assessment III [55], *n* = 31,188 to 32,315. Intake of added sugar in SSBs is for a 2000 kcal/day diet, compared with the recommended limit for the amount of added sugar recommended by the DGA (10% of calories), and by the Dietary Guidelines Advisory Committee (5% of calories) [56]; the amount consumed in SSBs alone for 48% of students was 96% the DGA limit of 10% of calories, and for 96% of students was 96% of the limit of 5% of calories [56]. I assumed 24.1 g of added sugar in a 12 oz SSB [59], and 4 kcal/gram. Adequacy of fruit and vegetable consumption in comparison to US Dietary Guidelines for Americans (DGA) recommended intake of 2 and 2.5 cups of fruit and vegetables per day, respectively [60], 83% had inadequate fruit, and 95% inadequate vegetable consumption. (**c**) Data from [58]; survey of students in 27 HEIs (including 22 PHEIs) in Minnesota, US, *n* = 13,720.

Therefore, the predominance of food security as the measure of student diet-related health diverts attention from the frequent finding that many food-secure students also have poor diets and health, although at lower prevalence than FI students. For example, a review of 16 studies found that FI students tend to have high intake of unhealthy and low intake of healthy foods, but with little difference from food-secure students, suggesting that all students have unhealthy diets [2]. One of the few studies on the health of the US college-age population found 56.3% overweight and obesity in 2017–2018, higher than most estimates of FI in this age group, and an increase from 23.9% in 1968–1970 [54] (Figure 4a). National College Health Assessment data show a high prevalence of FI similar to other campus surveys, but also show an equal prevalence of overweight/obesity, a higher prevalence of unhealthy levels of added sugar intake in the form of SSBs, and a much higher prevalence of inadequate fruit and vegetable intake [55] (Figure 4b). (Because SSBs comprise about one-third of added sugar intake for the college-age population [56], this level of consumption, in addition to added sugar in the rest of the diet, increases the risk of obesity and NCDs [57].) A survey of students at 27 HEIs (including 22 PHEIs) in Minnesota found 23.6% FI, but higher prevalence of frequent SSB intake, overweight and obesity, and much higher prevalence of inadequate fruit and vegetable intake for both food-secure and FI students, though somewhat higher for FI students [58] (Figure 4c).

Because food security is often the predominant, or only, indicator of diet-related student health used on campus, it diverts attention from unhealthy campus food environments. While these environments negatively affect the diets and health of all students, they pose a greater risk for FI students than for food-secure students. A survey of 1084 PHEI students, all with “unlimited meal plans and dining hall access” found that FI students had significant differences in diet compared with food-secure students, including 9% lower intake of both fruit and vegetables, and 56% higher intake of SSBs [61]. One cause may be that at low levels of FI, as worry about access to food and compromises on quality increase, the risk of overconsuming unhealthy food and of obesity can also increase [62]. These data help explain the frequent finding that FI is associated with overweight and obesity [49,58,63] (Figure 4c), although the “obesity-FI paradox” is more complicated [64].

### 2.3. Campus Food Environments, Student Food Security, and Health

Overall, the evidence suggests that unhealthy food environments on and near PHEI campuses are an important cause of the poor health of all students, but pose greater risks for FI students. Yet, as a result of the emphasis on FI as the indicator of diet-related student health, responses to FI focus on increasing the quantity of food available to individual FI students. However, increasing access to the food that currently dominates campus food environments would not improve the nutritional status of FI students, though it would decrease FI as currently measured, and can improve general health. For example, a survey of 1855 PHEI students, 60% of whom were FI, found that the use of campus food pantries was associated with student self-perception of improved health and sleep, and decreased depression [65], although this may be due not only to increased food access, but also to the efforts of many campus food pantries to improve the nutritional quality of food they provide [66].

There is also some evidence suggesting that it is the ability to successfully navigate the food environment, not only food access, that underlies student FI. For example, improving FI students’ food knowledge and skills can help them make healthier food choices and reduce the risk posed by an unhealthy food environment. A study of 171 undergraduates at a large PHEI enrolled in a nutrition and culinary skills class found that the course significantly decreased FI and stress levels, and that decreased FI was associated with increased vegetable and especially fruit intake [67] (although the mechanism for this increase was not investigated). FI students themselves have stated their desire for help in learning to cook inexpensive nutritious food [68].

While programs targeting individual FI students can decrease FI, and sometimes even improve health, they do not change the unhealthy campus food environments, which contribute to the poor diets and health of all students, both food-secure and FI. It seems as though addressing FI, as well as diet-related student health in general, is disconnected from the decisions that determine the unhealthy unsustainable PHEI campus food environments.

## 3. Why Are Campus Food Environments Unhealthy and Unsustainable? 

In the late 1930s and early 1940s in Europe and later the US, neoliberalism emerged as a self-identified movement characterized by assumptions favoring economic growth, markets, the private sector, and individual freedom, and in opposition to economic equity, social welfare, the public sector, and the public good [69]. Its negative effect on public health, via marketing of infant formula, was implicitly recognized 50 years ago by UCLA public health nutritionist Derrick Jelliffe as “commerciogenic malnutrition” [70]. 

Since then, neoliberalism’s role in creating food environments that profit food corporations and undermine public and planetary health has increased [9,15,71,72], although its negative role has been obscured by the dominance of neoliberalism in policies addressing both the public health crisis, e.g., in the social determinants of health policy frameworks [73], and the planetary health crisis, e.g., in promoting “green growth” based on the unsupported concept of absolute decoupling of economic growth from negative environmental impacts [74,75]. As a result, rather than question the assumptions underlying neoliberalism that have fueled the public and planetary health crisis, the most common approach to addressing this crisis is applying the neoliberal panacea of market-based solutions, technological fixes, and continued economic growth, with public–private partnerships frequently assumed to be inherently beneficial [76], which has become the norm in public health [77] and planetary health policy [71].

### 3.1. PHEIs, Neoliberalism, and Food

The rise of neoliberalism has led to the increasing privatization and marketization of higher education [22,23], with negative impacts on the public-good mission of PHEIs, including in the US [78]. Low income and BIPOC students have been disproportionately affected [3], for example, Latiné students’ basic needs in a PHEI Hispanic Serving Institution [4].

The neoliberalization of PHEIs has progressed to the point that it has become normalized and unremarkable [78,79]. For example, a business professor at a major PHEI was approvingly quoted in the official campus newsletter stating that the campus “has been underleveraging its brand” in marketing it to corporations that want “to have access to students who, after graduation, will continue to use their products and services” [80].

Neoliberalism dominates decisions about PHEI food environments that are largely made without the knowledge of or input from the campus community. These decisions entail agreements with food corporations, including purchasing contracts, dining hall food service contracts, leases of space to fast food corporations or their franchisees, PHEIs themselves becoming fast food franchisees, and revenue-generating contracts. Campuses are a major attraction for food corporations not only for current profits but for developing brand loyalty—as one corporate fast food franchising consultant stated “Start them young and hopefully they will be your customer for life” [81].

Revenue-generating contracts involve PHEIs being paid cash and commissions in return for giving the corporate partner the right to use the PHEI’s reputation in branding and product promotion, and exclusive rights to market their products to a captive audience of students [82,83]. These contracts also obligate PHEIs to collaborate with corporations in promoting their products. This type of contract increases the “depth to which companies are involved in student life” and their popularity with HEI administrators has grown dramatically in recent years [84].

The most common type of revenue-generating contract for US PHEIs is a beverage-pouring rights contract (PRC) with Coca-Cola or PepsiCo, which brings cash payments, commissions, and beverage promotion equipment to PHEIs, along with the obligation to collaborate with the corporation targeting students in the sale and promotion of sugar-sweetened beverages (SSBs) and other beverages [82,83]. 

In response to a request for PRCs from all 143 US PHEIs with 20,000 or more students in 2018–2019, researchers obtained 131 unique PRCs with either Coca-Cola or PepsiCo from 124 (87%) of those PHEIs. Of these contracts, 95% included at least one provision tying payments to sales volume, incentivizing the campus to promote sales of their corporate partner’s SSBs and other beverages to students [83], and 10% included provisions for paying students themselves to promote beverage sales to their peers [85]. While these contracts have a major effect on the campus food environment, students are not involved in decision making and are mostly unaware of these contracts. A survey at one PHEI found that only 21% of students were previously aware of their campus PRC [86].

PRCs reinforce the unhealthiness of campus food environments and student food choices. For example, on a PHEI campus with a PRC with PepsiCo of 940,773 thousand liters of beverages sold in one calendar year, SSBs comprised 66.8%, and first-year students consumed an average of 3658 g of added sugar per school year just in SSBs on campus alone (Meisterling et al. 2022). This level of added sugar consumption is equivalent to 30% of the USDA DGA, and 60% of the DGAC recommended maximum daily intake, yet does not include added sugar consumed in food on campus, or added sugar consumed in SSBs and food off campus [59].

The conflict of these contracts with PHEI’s public-good mission are particularly egregious because of the well-established negative health effects of SSBs and the unethical business practices of Coca-Cola and PepsiCo [6,87]. Some students are campaigning to end PRCs on their campuses because they see them as part of a neoliberal business policy that betrays their PHEI’s public-good mission [88], yet many students, as well as staff and faculty, also have a neoliberal perspective. For example, a survey of students, staff and faculty at one PHEI found that half supported PRCs, with support strongly associated with belief in individual responsibility for SSB consumption, and that half did not support PRCs, with lack of support strongly associated with belief that the campus environment is responsible for SSB consumption [86].

As Marion Nestle observed, PRCs “turn colleges and universities into pushers of sugary beverages…. Want to get pouring rights off of your campus? Good luck with that. This is a perfect example of money vs. public health. Guess which is more likely to win” [89].

### 3.2. PHEIs’ Public Good Mission and the Disconnect with Campus Food Environments

PHEIs and PHEI organizations have many public-good statements supporting public and planetary health, including on their public websites. For example, the American Association of State Colleges and Universities, with over 400 PHEI members, states that “Meeting the evolving challenges of today’s world demands that public colleges and universities creatively and effectively use their resources to serve the public good”, that partnerships with corporations and other entities should “support student learning and success”, and university leaders should “ensure that the partnership aligns with the institution’s mission” [90]. A major PHEI system states that it “values the health and wellbeing of its students, staff, faculty and… seeks to provide healthy and accessible conditions for the communities it serves, and this will be considered as a fundamental factor when making procurement decisions” [91].

Similar statements are made by large HEI organizations with many PHEI members. The Consortium of Universities for Global Health consisting of over 170 HEIs is dedicated to “addressing global health challenges” by “supporting academic institutions and partners to improve the wellbeing of people and the planet through education, research, service, and advocacy” [92]. The American College Health Association with over 800 HEI members has the core value of “promoting healthy campus communities and healthy individuals as integral to student learning” [55]. Over 600 North American HEIs have joined the Association for the Advancement of Sustainability in Higher Education (AASHE) “to lead the global sustainability transformation”, with sustainability defined as “encompassing human and ecological health, social justice, secure livelihoods and a better world for all generations” [93]. Hundreds of HEIs signed a Climate Emergency Letter that recognizes “the need for a drastic societal shift to combat the growing threat of climate change” [94].

**Figure 5:**
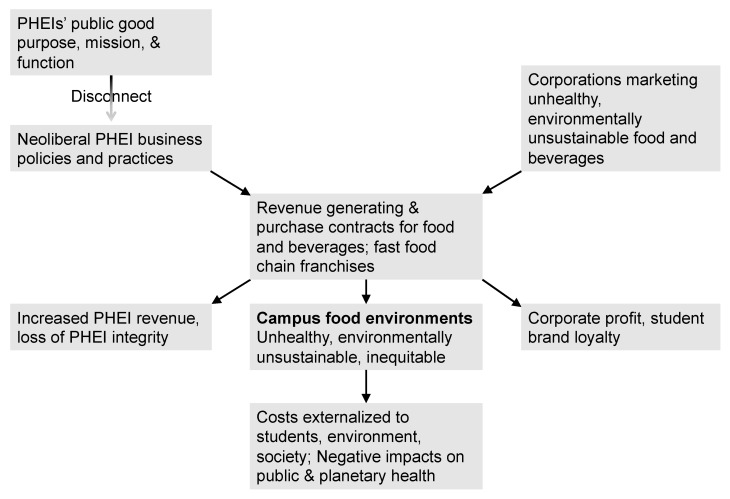
PHEI neoliberal policies drive unhealthy campus food environments and externalize negative impacts. © D.A. Cleveland, used with permission.

These statements reflect PHEIs’ purpose (what they should do), mission (what they say they do), and function (what society needs them to do) [76]. The disconnect between these and PHEIs’ neoliberal business policies that create unhealthy campus food environments results in a loss of institutional integrity [76] (Figure 5).

This disconnect is reflected in PHEI organizational structures, with decisions about food procurement at PHEIs usually being located in financial units that prioritize revenue generation and cost reduction and not in health, sustainability, or equity units that prioritize the public-good. This facilitates implementing neoliberal policies without having to directly or publicly confront the contradiction of these with the campus public-good purpose, mission or function, or the costs of the health, sociocultural, and environmental impacts of these decisions, which are externalized to students, the campus community, and beyond.

The loss of PHEI integrity affects experiences and attitudes across campus. PHEI students, staff and faculty trying to create healthier, more equitable campus food environments have noted the “challenge of dealing with campus structural constraints against enacting change, despite individual good intentions” because the campus treatment of “food as a cost-center…is…at odds with the wellness priority,” and the campus organizational system” “discourages equity and inclusion” [95]. Even when there are campus programs to reduce FI, the unhealthy food environments on campus send a very different message to students and undermine their ability to choose healthy foods, as well as their confidence that their campus cares about their health and well-being [38].

### 3.3. The FI Metric, Campus Food Environments, and Neoliberal Business Policies

Most research on student FI sponsored directly by PHEIs, PHEI organizations, and government bodies does not include the role of the food environment. For example, the National College Health Assessment asks students questions about FI, diet, weight, and campus social environments, but not about campus food environments [55]. PHEI policies to mitigate FI reflect this research approach by focusing on individual students and food quantity, e.g., with food pantries, meal plan pass donations, increasing access to SNAP (Supplemental Nutrition Assistance Program), and financial aid, as documented, e.g., in a review of studies of 58 HEIs (88% PHEIs) [96]. The same is true for federal programs that target student FI [51], which emphasize access to SNAP [50]. While some recommendations include measures, such as campus gardens and farmers’ markets [97], these would have little effect on the dominant campus food environment. More systemic solutions to the resource inequity that drives FI, including PHEI advocacy for policies that would redistribute wealth, are not feasible due to the dominance of neoliberal ideology [78].

However, many PHEI staff in programs to reduce FI are well aware of the limitations of simply increasing food quantity, and work to increases nutrition security, for example, by offering healthier food and cooking and nutrition information in food pantries. A 2021 survey of 352 HEI food pantries (75% at PHEIs) found 52% offered fresh fruits and vegetables, 27% sourced food from farmers’ markets and community gardens, and 33% offered cooking classes and/or nutrition information [66]. However, although campus food pantries were originally created as an emergency measure, they appear to have become a long-term solution, yet have to continually seek funding, which is often inadequate [65], resulting in major obstacles, such as a lack of space and refrigeration for fresh fruit and vegetables [66]. However, these programs also address the problem at the individual level and are not able to include the campus food environment [97].

The cost of FI and poor student health falls on students in the form of mental stress, academic anxiety, and NCDs, and directly and indirectly on their families and society. FI is especially critical for students because it is associated with poor academic performance [49,68,98]. These costs are borne disproportionately by students from low-income and BIPOC communities [49,58,97,98], the communities targeted by junk food and beverage advertising [87,99,100], and comprise an increasing proportion of HEI students over the last 20 years [100,101], reaching 43.4% in fall 2019 [24]. These costs are not considered in estimating the benefit of PHEI financial decisions about food on campus. In contrast, mission statements and programs aimed at reducing student FI and supporting student health have little influence on campus food environments, but are much more widely publicized than neoliberal business policy.

## 4. How Can PHEIs Transform Campus Food Environments Commensurate with the Unprecedented Challenge of the Public and Planetary Health Crisis?

**Figure 6:**
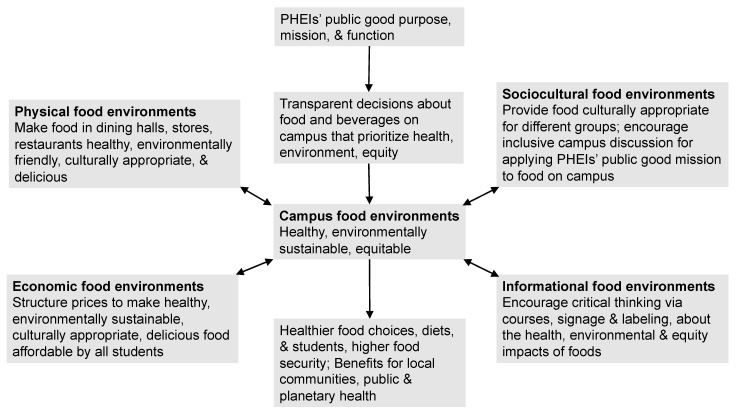
Aligning campus food policy with PHEIs’ public-good purpose, mission and function to transform campus food environments. © D.A. Cleveland, used with permission.

A comprehensive campus food policy aligned with PHEIs’ public-good purpose, mission, and function would address all four components of the food environment in responding to student FI and poor health, as well as the public and planetary health crisis (Figure 6).

### 4.1. Past Approaches Limited by Neoliberalism

There are many examples of limited positive changes in some components of PHEI food environments, especially in residential dining halls, because they are relatively more insulated from market competition and the risk of reduced revenue, due to having a captive clientele of mostly first-year students. Student activists have been successful in pressuring PHEI administrators to switch from large food service corporations, who may prioritize profit [102], to self-operated dining halls [103]. Many PHEIs are increasing the proportion of plant-based foods in dining halls as a result of student and staff advocacy [104,105], which could improve student health and reduce environmental impact from animal agriculture and reduced healthcare costs [105]. The self-operated residential dining of one large PHEI worked with a local food hub to successfully increase fresh, local, and organic produce in dining halls by prioritizing social and environmental goals, although without compromising financial goals [106]. However, while changes such as these do challenge neoliberal business policies to some extent, their effects on the general campus food environment outside of dining halls, are limited.

General campus food environments have also seen some incremental improvements as a result of student activism, e.g., in forcing administrators to not renew PRCs at two PHEIs, and faculty activism in forcing a ban on the sale of SSBs at a large PHEI [107]. However, the dominance of neoliberalism limits the extent of change. A key limitation is that these changes do not address the need to reduce superfluous consumption, which is an essential component of an adequate response to the public and planetary health crisis [12,13,14,15]. Instead, the dominance of neoliberalism means that not reducing overall revenue (consumption) is considered a benefit of these limited changes. As a result, sales of more healthy, more environmentally sustainable, but superfluous food are often expected to replace revenue lost from decreasing sales of less healthy, less environmentally sustainable food, for example, by promoting sales of non-SSB beverages, including bottled water, when SSB consumption is reduced, instead of promoting tap water consumption [59].

One PHEI’s healthy beverage initiative was considered “viable” because a decrease in SSB sales was compensated for by an increase in non-SSB beverage sales, even though increasing tap water consumption was part of the initiative, which, if successful, would reduce beverage sales [108]. This means that environmental impact likely did not change much, whereas if tap water replaced SSBs, it would greatly reduce the environmental impact from beverages and beverage containers [59]. Improving food environments could even lead to increased net consumption—a healthy food policy at a large PHEI increased vending machine sales of “healthier” snacks and beverages by 5% and 4%, respectively in 2018–2019, while total sales increased by 12% and 15% [109], although total student fall enrollment increased only 1.6% [110].

An important potential driver of increased consumption on campus is the many new “healthy” and “sustainable” beverage and “alternative protein food” startups that seek large profits from increased consumer preference for more healthy environmentally sustainable food [111]. By proposing the neoliberal solution of addressing “market failures” by reducing some negative health and environmental externalities of food while increasing revenue and consumption, contracts with these corporations may appeal to PHEI administrators [112]. These products can decrease the negative health and environmental impacts per unit of food, yet, because the companies are funded by private equity and venture capital, they aggressively prioritize increasing sales, and profit. Therefore, contracts with these companies can encourage superfluous consumption, and undermine public and planetary health and the public-good mission of PHEIs, as do contracts with private online education companies [113]. Adequately addressing the crisis of student, public and planetary health will require more fundamental change, including a move away from superfluous consumption to sufficiency [15].

### 4.2. Beyond Neoliberalism

An important beginning step in transforming campus food environments will be moving basic decisions about campus food from financial units, such as procurement, to independent units, where decisions aligned with campus public-good policies would be made by representatives of students, staff, and faculty. This will be challenging, but a step in this direction has been made by a hospital, where “the desire to reorient foodservice to center public health required significant change in the organizational roles and structures [which had] supported a revenue-centric mission at the expense of public health” [114]. However, for such changes to come about and adequately improve the food environment more fundamental systemic change is also required.

Systemic changes in campus food environments commensurate with the challenge of the public and planetary health crisis are only likely to come about following inclusive, deep, transparent discussion on campus. This will not be easy because the normalization of neoliberalism in PHEIs, with the marketization of everything, including PHEIs’ reputations and brands, “crowds out” discourse about the public good, reducing discussions to technocratic, economistic terms in which markets are erroneously assumed to deliver neutral answers [115].

Successful discussions will require faculties willing to translate knowledge of the crisis into action at their PHEIs [116]. This includes listening to students [4], as well as supporting their activism, and working with them to understand the implications of food choices and food environments, for example, in course work. This means creating an educational context that engages students’ values and knowledge in ways that empower their critical thinking about food and its effects on individuals, society, and the environment [117]. A course at one PHEI on the environmental impact of student diets resulted in students making a significant reduction in meat consumption with a corresponding reduction in climate impact [118].

However, the main goal of campus discussion and advocacy will need to be convincing campus administrators to move beyond neoliberal logic and implement the campus public-good mission in the food environment. Therefore, acknowledgement of and “systematic engagement” with the neoliberal assumptions [71,72] driving PHEI business policies is needed, including the moral values embedded in those assumptions [69,115,119], followed by discussion of how to prioritize public-good values and policies that respond adequately to the public and planetary health crisis, e.g., degrowth instead of economic growth, ecological economics instead of neoclassical economics, and sufficiency instead of superfluous consumption [15,112,120].

## 5. Conclusions

More research on the relationship among and between campus food environments and student health, well-being, food security, and academic success on one hand, and campus business, health, sustainability, and equity policies on the other hand, is needed to clarify the mechanisms, causality, and strength of relationships in different contexts. However, my narrative review of salient literature strongly suggests that the broad outline of the determinants of PHEI campus food environments and their effects are well known—neoliberalism drives food environments that contribute to student FI and poor health, and to the public and planetary health crisis, and leads to the loss of PHEI integrity.

Instead, PHEIs can inspire their students and the world by rejecting neoliberalism and embracing a public good response to the public and planetary health crisis. Indeed, PHEIs have the potential and the responsibility, as key research and educational institutions with a public-good mission, to lead in responding to the diet-related student health crisis, but also to the public and planetary health crisis in which student health is embedded. This includes aligning their food, beverage, and other policies, with their public-good purpose, mission, and function, including reducing superfluous consumption. The current challenge to the global dominance of neoliberalism by a populist libertarian backlash, may present an opportunity and incentive for radically rethinking PHEIs [121] in ways that honor their public-good mission and adequately respond to the crisis. This will require inclusive campus and community discussion about the policy changes needed for this response, and how to achieve them. My goal in this perspective is to encourage this discussion.

## Data Availability

Not applicable.
